# Associations of the fasting triglyceride glucose index with pulse wave velocity vary by age and gender

**DOI:** 10.20945/2359-4292-2025-0115

**Published:** 2025-08-18

**Authors:** Yen-Fu Chen, Yi-Chih Chang, Wen-Cheng Li, Po-Ya Lin, Yi-Hsuan Chen, Yi-Chuan Chen, Ting-An Yang, Jo-Hsuan Chen

**Affiliations:** 1 Department of Family Medicine, Chang-Gung Memorial Hospital at Linkou, Taoyuan, Taiwan; 2 Department of Cardiology, Xiamen Chang-Gung Hospital, Xiamen, China; 3 College of Medicine, Chang Gung University, Taoyuan, Taiwan

**Keywords:** Atherosclerosis, triglyceride glucose index, pulse wave velocity, gender, age

## Abstract

**Objective:**

This study determined the optimal cutoff point for the triglyceride-glucose (TyG) index for predicting subclinical atherosclerosis (SA). Subjects and

**methods:**

Overall, 10,039 participants (5,598 men and 4,441 women) aged > 18 years were recruited from Xiamen Chang Gung Hospital. Demographic information was provided, and the TyG index was calculated. The TyG index was categorized into quartiles, and SA was assessed by measuring brachial-ankle pulse wave velocity (baPWV). The cutoff point for the TyG index was determined via receiver operating characteristic (ROC) curve analysis.

**Results:**

SA incidence increased with increasing TyG index in both men (from 5.929% in Group I to 10.579% in Group IV; P < 0.001) and women (from 2.074% in Group I to 14.955% in Group IV; P < 0.001). Multivariate linear regression analysis revealed that a higher TyG index was associated with an elevated risk of SA in men (odds ratio [OR] 4.028, 95% confidence interval [CI] 2.811-5.711) and women (OR 2.599, 95% CI 1.86-5.543). ROC curve analysis revealed that the area under the curve was 0.572 (95% CI = 0.541-0.602; P < 0.001) for men and 0.694 (95% CI = 0.668-0.721; P < 0.001) for women. The optimal TyG index cutoff points for predicting subclinical atherosclerosis were 8.961 for men (sensitivity, 46.5%; specificity, 67.9%) and 8.254 for women (sensitivity, 79.7%; specificity, 49.9%).

**Conclusion:**

The TyG index is a composite indicator of dyslipidemia and hyperglycemia. In clinical practice, women with TyG index values above the cutoff should be further evaluated for the underlying pulse wave velocity.

## Introduction

The interplay between metabolic dysfunction and cardiovascular health is a key area of medical research with the potential to improve diagnostic and treatment strategies. Among the various indicators used to assess metabolic health, the triglyceride-glucose (TyG) index has attracted significant attention. The TyG index, which is derived from fasting triglyceride and blood glucose levels, serves as a surrogate marker for insulin resistance (IR), which is a fundamental mechanism in the pathogenesis of metabolic syndrome and type 2 diabetes mellitus (T2DM) (^1,2^).

Pulse wave velocity (PWV) is another important indicator in cardiovascular medicine that represents the speed at which blood pressure (BP) waves travel through arterial trees. It is a direct measure of arterial stiffness and is considered a reliable predictor of cardiovascular events and overall cardiovascular health. Elevated PWV values indicate increased arterial stiffness, which is associated with increased risks of hypertension, atherosclerosis, and other cardiovascular diseases (CVDs) (^3,4^).

Understanding the relationship between the TyG index and the PWV is critical for several reasons. First, both parameters are relatively easy to measure and provide important insights into metabolic and cardiovascular health. Second, elucidating this relationship may enhance our ability to predict cardiovascular risk in patients with metabolic diseases, thereby improving preventive strategies and therapeutic interventions. Several studies have demonstrated that higher TyG index values are associated with increased arterial stiffness, as indicated by higher PWV measurements (^5,6^). This correlation suggests that insulin resistance, as reflected by the TyG index, may contribute to the development and progression of arterial stiffness. However, the precise mechanisms linking these two parameters remain a subject of ongoing research. The potential pathways include chronic inflammation, oxidative stress, and endothelial dysfunction, all of which are exacerbated by insulin resistance, leading to vascular damage and stiffness (^7^).

The aim of this study was to enhance our understanding of the interrelationships between cardiometabolic risk factors - particularly the triglyceride-glucose (TyG) index - pulse wave velocity (PWV) and subclinical arterial stiffness (SA). This relationship may help identify novel biomarkers that support early detection and enable sex-specific risk stratification in clinical practice. By exploring the optimal TyG index threshold, we aim to facilitate early identification of high-risk individuals and inform targeted interventions to mitigate cardiovascular risk.

## SUBJECTS AND METHODS

### Population

This cross-sectional observational study included adults aged ≥ 18 years who attended annual health examinations at Xiamen Chang-Gung Hospital from 2013 to 2015. This study was approved by the Institutional Review Board of Xiamen Chang-Gung Hospital and conducted in accordance with the guidelines of the Declaration of Helsinki.

### Inclusion criteria

The participants were required to have comprehensive medical records, including medical and medication histories. During the physical examination, the participants fasted for > 12 h, and the women were not pregnant. The health examination parameters included height, weight, waist circumference (WC), BP, total cholesterol (TC), low-density lipoprotein cholesterol (LDL-C), high-density lipoprotein cholesterol (HDL-C), triglyceride (TG), fasting glucose, brachial-ankle pulse wave velocity (baPWV), and the ankle-brachial index (ABI).

### Exclusion criteria

Patients were excluded if they had any of the following conditions: 1. Chronic diseases that could significantly affect metabolism, such as thyroid dysfunction or chronic hepatitis. 2. Current use of hypoglycemic drugs or steroids that affect metabolism.

### Data collection and measurements

During the health examination, the participants were surveyed regarding their medical history, including any previous diseases or medication usage. Trained nurses followed standard operating procedures to collect venous blood samples and administered questionnaires to collect the data.

The clinical chemistry workup included various measurements conducted according to the standardized procedures of a hospital laboratory accredited by the College of American Pathologists. Clinical biochemistry tests included the measurement of fasting plasma glucose levels via a modified hexokinase enzymatic assay (Cobas Mira Chemistry System; Roche Diagnostic Systems, Montclair, New Jersey, USA). Fasting glucose, total cholesterol (TC), low-density lipoprotein cholesterol (LDL-C), high-density lipoprotein cholesterol (HDL-C), and triglyceride (TG) levels were measured via a biochemical autoanalyzer (DxC 800; Beckman Coulter UniCel DxC SYNCHRON, Ireland). Accurate measurements were obtained via calibrated instruments.

Additionally, the participants’ BP, height, weight, and WC were measured via calibrated instruments. BP was recorded three times via an automated sphygmomanometer, with measurements taken after the participant had been seated for at least 15 min. The mean arterial pressure (MAP) was estimated via the following equation: 2/3 × diastolic pressure + 1/3 × systolic pressure. Height and weight were measured to the nearest 0.1 cm and 0.1 kg, respectively. Body mass index (BMI) was calculated by dividing weight in kilograms by the square of height in meters (kg/m^2^).

baPWV was measured via an ABI-form device (VP1000, Colin Co. Ltd., Komaki, Japan), which enables simultaneous measurement of systolic BP and pulse waves of the brachial and posterior tibial arteries in all four extremities. The average baPWV and ankle-brachial index (ABI) were calculated for each individual. After resting for > 5 min in the supine position, four cuffs were wrapped around the bilateral brachia and ankles and connected to a plethysmographic sensor and an oscillometric pressure sensor. The ABI was measured by dividing the ankle systolic blood pressure (SBP) by the brachial SBP. Pressure waveforms were recorded via semiconductor pressure sensors to assess the transmission time between the initial rise in both brachial and tibial artery waves. The distance between the baPWV sampling points was estimated based on height. The baPWV was calculated via the formula (La - Lb)/Tba, where La is the distance from the heart to the ankle, Lb is the distance from the heart to the brachium, and Tba is the time interval between the brachial and ankle waveforms. The measurements were performed twice by trained technicians, and the average values of the leftand right-sided assessments were used to identify arterial stiffness markers.

### Definition of SA

SA was defined as a mean baPWV > 1,700 cm/s (^8^).

### Definition of the TyG index

The TyG index was calculated via the following formula: ln[fasting triglyceride (mg/dL) × fasting plasma glucose (mg/dL)/2] (^9^).

### Statistical analysis

Parametric continuous variables are expressed as the means ± standard deviations. Categorical data are expressed as numbers (percentages). Differences were tested via the chi-square test for categorical variables, Student’s t test for normally distributed continuous variables, and the Mann-Whitney U test for nonnormally distributed variables. Differences between the groups were assessed via the chi-square test for categorical variables and one-way analysis of variance (ANOVA) for continuous variables. Pairwise post hoc comparisons were performed via Bonferroni adjustment when the overall relationship was significant.

The relationships between risk factors for subclinical atherosclerosis and TyG index quartiles were examined via univariate and multivariate logistic regressions. The results are presented as odds ratios (ORs) with 95% confidence intervals (CIs). Receiver operating characteristic (ROC) curves for subclinical atherosclerosis and the TyG index were generated to determine the cutoff point value and evaluate the predictive power.

All the statistical analyses were performed via SPSS version 26.0 (SPSS, Armonk, NY, USA). Statistical significance was defined as a two-sided P value of < 0.05.

## RESULTS

Table 1 shows that the BMI, waist-to-height ratio (WHtR), MAP, fasting glucose, TC, TG, LDL-C, TG/HDL-C ratio, PWV, ABI, and TyG index, and HDL cholesterol levels are significantly greater in males than in females.

**Table 1:** Main characteristics of the study participants by sex

Characteristics	Total	Men	Women	P value
Number of subject	10,039	5,598	4,441	
Age, years	47.61 ± 10.36	47.411 ± 10.250	47.864 ± 10.483	0.030
BMI (kg/m^2^)	23.83 ± 3.30	24.470 ± 3.205	23.026 ± 3.244	<0.001
Waist-to-height ratio (cm/cm)	0.51 ± 0.06	0.512 ± 0.051	0.499 ± 0.060	<0.001
Mean arterial pressure (mmHg)	87.3 ± 13.30	90.139 ± 12.830	83.731 ± 13.015	<0.001
Fasting glucose (mmol/L)	5.33 ± 1.34	5.435 ± 1.505	5.190 ± 1.082	<0.001
Total cholesterol (mmol/L)	5.21 ± 0.98	5.266 ± 0.965	5.138 ± 0.989	<0.001
Triglycerides (mmol/L)	1.55 ± 1.41	1.818 ± 1.546	1.204 ± 1.124	<0.001
LDL cholesterol (mmol/L)	3.31 ± 0.85	3.424 ± 0.850	3.175 ± 0.835	<0.001
HDL cholesterol (mmol/L)	1.32 ± 0.31	1.213 ± 0.277	1.446 ± 0.306	<0.001
TG / HDL-C	1.36 ± 1.76	1.688 ± 1.953	0.937 ± 1.380	<0.001
PWV	1338.73 ± 265.51	1363.659 ± 264.625	1307.313 ± 263.313	<0.001
ABI	1.13 ± 0.09	1.140 ± 0.088	1.109 ± 0.079	<0.001
TyG index	8.57 ± 0.66	8.750 ± 0.661	8.340 ± 0.583	<0.001
SA	754 (7.5%)	400 (7.1%)	354 (8.0%)	0.119

Table 2 illustrates the associations between the TyG index and the characteristics of the study population. The participants were categorized into four quantiles of the TyG index (from smallest to largest). Among males, there were significant increasing trends in BMI, WHtR, MAP, fasting glucose, total cholesterol, triglycerides, the TG/HDL-C ratio, PWV, and subclinical atherosclerosis from TyG Q1 to Q4, accompanied by a significant decreasing trend in HDL cholesterol. Similarly, among females, there was a significant increasing trend in BMI, WHtR, MAP, fasting glucose, TC, TG, LDL cholesterol, the TG/HDL-C ratio, PWV, ABI, and SA from TyG Q1 to Q4 and a declining trend in HDL cholesterol.

**Table 2:** General characteristics of the study population according to sex-specific TyG index

	TyG					
Men	TyG Q1	TyG Q2	TyG Q3	TyG Q4	P value	P trend
Number	1,400	1,399	1,400	1,399		
Age, years	47.471 ± 11.532	48.149 ± 10.386	47.079 ± 9.560	46.946 ± 9.344	0.009	0.031
BMI (kg/m^2^)	22.556 ± 2.886	24.070 ± 2.948^a^	25.224 ± 2.893^a,b^	26.032 ± 2.981^a,b,c^	<0.001	<0.001
Waist-to-height ratio (cm/cm)	0.481 ± 0.048	0.506 ± 0.047^a^	0.523 ± 0.045^a,b^	0.537 ± 0.044^a,b,c^	<0.001	<0.001
Mean arterial pressure (mmHg)	85.273 ± 11.576	89.143 ± 12.258^a^	91.222 ± 12.570^a,b^	94.922 ± 12.955^a,b,c^	<0.001	<0.001
Fasting glucose (mmol/L)	4.888 ± 0.458	5.120 ± 0.623^a^	5.343 ± 0.942^a,b^	6.389 ± 2.503^a,b,c^	<0.001	<0.001
Total cholesterol (mmol/L)	4.860 ± 0.854	5.196 ± 0.868^a^	5.383 ± 0.925^a,b^	5.627 ± 1.034^a,b,c^	<0.001	<0.001
Triglycerides (mmol/L)	0.782 ± 0.172	1.219 ± 0.175^a^	1.757 ± 0.300^a,b^	3.515 ± 2.259^a,b,c^	<0.001	<0.001
LDL cholesterol (mmol/L)	3.137 ± 0.769	3.512 ± 0.776^a^	3.620 ± 0.832^a,b^	3.427 ± 0.937^a,c^	<0.001	<0.001
HDL cholesterol (mmol/L)	1.381 ± 0.305	1.249 ± 0.250^a^	1.160 ± 0.230^a,b^	1.061 ± 0.208^a,b,c^	<0.001	<0.001
TG/HDL-C	0.597 ± 0.195	1.016 ± 0.258^a^	1.580 ± 0.439^a,b^	3.561 ± 3.132^a,b,c^	<0.001	<0.001
PWV	1325.569 ± 263.273	1357.543 ± 256.734^a^	1355.902 ± 221.336^a^	1415.656 ± 302.987^a,b,c^	<0.001	<0.001
ABI	1.138 ± 0.085	1.141 ± 0.088	1.143 ± 0.092	1.139 ± 0.086	0.461	0.592
TyG	7.989 ± 0.240	8.494 ± 0.115^a^	8.893 ± 0.125^a,b^	9.623 ± 0.487^a,b,c^	<0.001	<0.001
SA	83 (5.929%)	87 (6.219%)	82 (5.857%)	148 (10.579%)^a,b,c^	<0.001	<0.001
Women	TyG Q1	TyG Q2	TyG Q3	TyG Q4	P value	P trend
Number	1,109	1,111	1,111	1,110		
Age, years	42.740 ± 9.415	46.322 ± 9.928^a^	49.748 ± 10.193^a,b^	52.641 ± 9.682^a,b,c^	<0.001	<0.001
BMI (kg/m^2^)	21.236 ± 2.529	22.403 ± 2.896^a^	23.536 ± 3.128^a,b^	24.926 ± 3.176^a,b,c^	<0.001	<0.001
Waist-to-height ratio (cm/cm)	0.464 ± 0.049	0.487 ± 0.054^a^	0.508 ± 0.056^a,b^	0.536 ± 0.056^a,b,c^	<0.001	<0.001
Mean arterial pressure (mmHg)	78.024 ± 10.580	81.557 ± 12.253^a^	85.385 ± 12.801^a,b^	89.954 ± 13.161^a,b,c^	<0.001	<0.001
Fasting glucose (mmol/L)	4.804 ± 0.412	4.977 ± 0.486^a^	5.116 ± 0.506^a,b^	5.865 ± 1.836^a,b,c^	<0.001	<0.001
Total cholesterol (mmol/L)	4.710 ± 0.835	5.005 ± 0.881^a^	5.286 ± 0.889^a,b^	5.549 ± 1.124^a,b,c^	<0.001	<0.001
Triglycerides (mmol/L)	0.574 ± 0.118	0.853 ± 0.105^a^	1.183 ± 0.165^a,b^	2.206 ± 1.867^a,b,c^	<0.001	<0.001
LDL cholesterol (mmol/L)	2.762 ± 0.675	3.096 ± 0.739^a^	3.372 ± 0.775^a,b^	3.472 ± 0.940^a,b,c^	<0.001	<0.001
HDL cholesterol (mmol/L)	1.606 ± 0.307	1.514 ± 0.279^a^	1.421 ± 0.267^a,b^	1.243 ± 0.243^a,b,c^	<0.001	<0.001
TG/HDL-C	0.371 ± 0.107	0.583 ± 0.135^a^	0.865 ± 0.221^a,b^	1.927 ± 2.472^a,b,c^	<0.001	<0.001
PWV	1191.580 ± 188.559	1260.315 ± 242.925^a^	1345.812 ± 267.919^a,b^	1431.449 ± 280.421^a,b,c^	<0.001	<0.001
ABI	1.097 ± 0.080	1.105 ± 0.082	1.114 ± 0.075^a,b^	1.120 ± 0.077^a,b^	<0.001	<0.001
TyG	7.666 ± 0.226	8.113 ± 0.100^a^	8.466 ± 0.111^a,b^	9.113 ± 0.411^a,b,c^	<0.001	<0.001
SA	23 (2.074%)	63 (5.671%)^a^	102 (9.181%)^a,b^	166 (14.955%)^a,b,c^	<0.001	<0.001

a p < 0.05 versus TyG Q1;

b p < 0.05 versus TyG Q2;

c p < 0.05 versus TyG Q3.

Table 3 presents the associations between the TyG index and SA. In males, the prevalence of SA was 5.929%, 6.219%, 5.857%, and 10.579% for Q1, Q2, Q3, and Q4 of the TyG index, respectively. For females, the prevalence rates were 2.074%, 5.671%, 9.181%, and 14.955% for Q1, Q2, Q3, and Q4, respectively. After adjusting for age, HDL-C, and LDL-C, a higher odds ratio of SA was associated with a higher TyG index, comparing Q4 and Q3 to Q1 in males and Q4, Q3, and Q2 to Q1 in females.

**Table 3:** Unadjusted and adjusted odds ratios with 95% confidence intervals for subclinical atherosclerosis in men and women

Men	n, (%)	SA										
		Model 1				Model 2				Model 3		
		OR	(95% CI)	P value		OR	(95% CI)	P value		OR	(95% CI)	P value
TyG Q1	83 (5.929%)		reference				reference				reference	
TyG Q2	87 (6.219%)	1.052	0.771 to 1.435	0.748		1.145	0.815 to 1.609	0.434		1.312	0.922 to 1.868	0.132
TyG Q3	82 (5.857%)	0.987	0.721 to 1.352	0.936		1.360	0.963 to 1.922	0.081		1.700	1.170 to 2.469	0.005
TyG Q4	148 (10.579%)	1.877	1.419 to 2.483	<0.001		3.015	2.197 to 4.137	<0.001		4.028	2.811 to 5.771	<0.001
P trend			<0.001				<0.001				<0.001	
Women	n, (%)	Model 1				Model 2				Model 3		
		OR	(95% CI)	P value		OR	(95% CI)	P value		OR	(95% CI)	P value
TyG Q1	23 (2.074%)		reference				reference				reference	
TyG Q2	63 (5.671%)	2.838	1.748 to 4.610	<0.001		1.859	1.086 to 3.183	0.024		1.848	1.109 to 3.284	0.020
TyG Q3	102 (9.181%)	4.773	3.012 to 7.565	<0.001		2.018	1.213 to 3.355	0.007		2.054	1.258 to 3.627	0.005
TyG Q4	166 (14.955%)	8.303	5.322 to 12.953	<0.001		2.891	1.772 to 4.717	<0.001		2.599	1.860 to 5.543	<0.001
P trend			<0.001				<0.001				<0.001	

Model definitions are: Model 1, unadjusted analysis; Model 2, adjusted for age; Model 3, Model 2 + HDL-C, and LDL-C level

**Figure 1:**
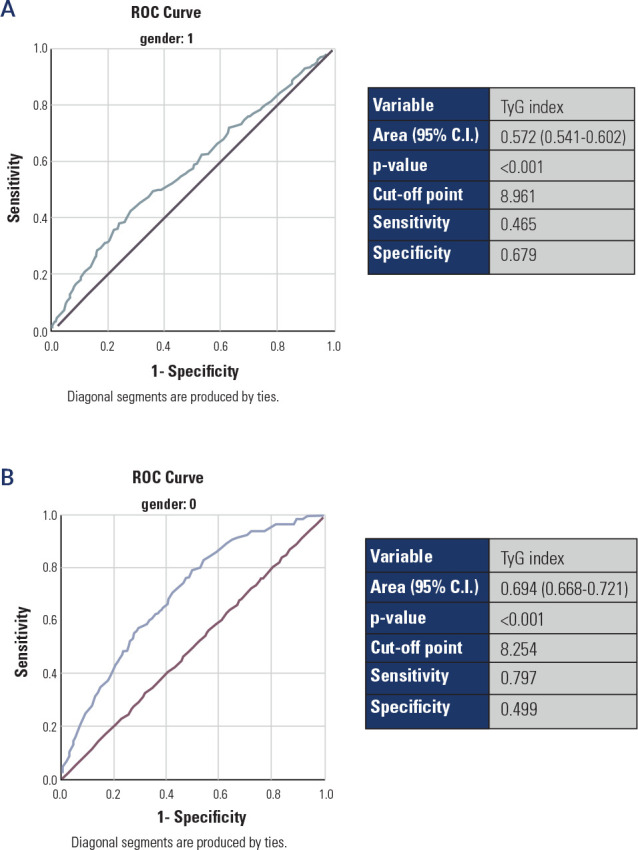
**(A)**. Receiver Operating Curve (ROC) analyses of the TyG index as a predictor of subclinical atherosclerosis in men; (**B**) ROC analyses of the TyG index as a predictor of subclinical atherosclerosis in women

As shown in **Figure 1**, according to the ROC curve analysis, the AUCs were 0.572 (95% CI: 0.541-0.602, specificity 67.9%, sensitivity 46.5%) for men (**Figure 1A**) and 0.694 (specificity 49.9%, sensitivity 79.7%) for women (**Figure 1B**). Furthermore, the optimal cutoff point of the TyG index for predicting SA incidence was 8.961 for males and 8.254 for females.

## DISCUSSION

This cross-sectional study further highlights the association between the TyG index and SA. Our study has three main findings. First, higher PWV rates and SA were associated with increased TyG quartiles in both men and women, whereas increased ABI was associated with higher TyG quartiles only in women. Second, even after adjusting for age and HDL-C and LDL-C levels, the odds of developing SA were significantly greater in the highest TyG quartile than in the lowest quartile. Third, the TyG index cutoff showed greater validity in predicting SA in women. Therefore, in clinical practice, women with TyG index values above the cutoff should be further evaluated for underlying SA.

### TyG Index, BMI, and WHtR

IR is a driving factor for nonalcoholic fatty liver disease and coronary heart disease (CHD). Research indicates that insulin plays a critical role in maintaining vascular tone by exerting nitric oxide (NO)-dependent vasodilation and endothelin-1 (ET-1)-dependent vasoconstrictive effects through the phosphatidylinositol 3-kinase (PI3K) and mitogen-activated protein kinase (MAPK) pathways. At physiological concentrations, insulin maintains the balance between these pathways. However, IR disrupts this balance by impairing the PI3K-NO pathway and enhancing the MAPK-ET-1 pathway, leading to endothelial dysfunction (^10,11^). Endothelial dysfunction is also associated with glucose and lipid toxicity resulting from metabolic abnormalities. These abnormal physiological processes are particularly common in patients with obesity and are related primarily to the activation and accumulation of macrophages. An important pathway by which obesity induces low-grade inflammation involves the activation and migration of macrophages, which release various inflammatory factors, such as interleukins and tumor necrosis factor (^12^). These factors create an inflammatory environment that impedes the action of insulin on adipocytes, leading to IR. The systemic inflammatory response in obese patients is closely linked to CHD (^13^). In our study, both BMI and WHtR in men and women increased significantly with increasing TyG index quartiles. A 15-year prospective study of an urban Chinese population revealed strong correlations among the TyG index, BMI, and components of metabolic syndrome (^14^). Another study emphasized that the TyG index is more effective in predicting IR than traditional indicators such as BMI or WHtR. These findings suggest that combining the TyG index with BMI or WHtR can better predict metabolic disorders (^15^).

### TyG Index and BP

IR and SA complications are believed to be associated with adipokines, including dysregulation of tumor necrosis factor-alpha. This dysregulation is linked to reduced NO production in vascular endothelial cells, thereby promoting atherosclerosis (^16^). The TyG index is associated with hypertension, possibly through hyperinsulinemia related to IR, which increases sympathetic nervous system activity or activates the renin-angiotensin-aldosterone system (^17^). In this study, the MAP increased significantly with higher quartiles of the TyG index in both men and women. A population-based study investigating the relationship between the TyG index and BP in individuals with normal BP revealed a significant correlation, suggesting that the TyG index can be used to identify individuals at risk of hypertension (^18^). Another study analyzing the association between the TyG index and central systolic pressure in adult patients with hypertension revealed that a higher TyG index was associated with increased central systolic pressure, highlighting its role in predicting BP-related cardiovascular risk (^19^). A meta-analysis exploring the association between the TyG index and hypertension revealed that individuals with higher TyG index values had a significantly increased risk of developing hypertension, underscoring its potential as a marker of hypertension risk (^20^).

### TyG index and subclinical atherosclerosis

The TyG index is a composite indicator of dyslipidemia and hyperglycemia, both of which are critical factors in the development of atherosclerosis. The relationship between the TyG index and SA can be understood through multiple mechanisms. High TyG index values indicate IR, leading to endothelial dysfunction and inflammation (^21^). Second, elevated triglyceride and glucose levels increase oxidative stress, resulting in lipid peroxidation and vascular damage (^22^). Finally, hyperglycemia and dyslipidemia impair endothelial function and reduce NO utilization, leading to vascular stiffness and atherosclerosis (^23^).

In our study, higher PWV rates and SA in both men and women were associated with higher TyG index quartiles. A review from Japan examining the relationship between various IR indices and SA revealed a positive correlation with the TyG index, indicating that individuals with higher TyG indices have a greater risk of developing atherosclerosis (^24^). The TyG index, as a predictor of SA in patients without diabetes, revealed a strong association, emphasizing its utility for early detection and risk stratification (^25^). A meta-analysis evaluating the TyG index as a marker of SA and arterial stiffness confirmed its association with a greater likelihood of SA (^26^).

### Gender differences in the TyG index and subclinical atherosclerosis

Our findings revealed a stronger association between the TyG index and subclinical atherosclerosis (SA) in women than in men, suggesting the potential utility of the TyG index as a sex-specific marker for early cardiovascular risk stratification. Several biological mechanisms may underlie this observed disparity:

Hormonal regulation: Estrogen, particularly in premenopausal women, exerts vasoprotective effects by enhancing endothelial function, favorably modulating lipid metabolism, and improving insulin sensitivity. These mechanisms contribute to lower TyG index values and may attenuate the progression of atherosclerosis in women (^27^). In contrast, men exhibit higher circulating testosterone levels, which have been linked to atherogenic lipid profiles - characterized by elevated triglycerides and reduced HDL cholesterol - thereby increasing TyG index values and the likelihood of SA (^28^).

Adipose Tissue Distribution: Men are more prone to visceral fat accumulation, which is metabolically active and strongly associated with insulin resistance and systemic inflammation. This pattern correlates with higher TyG index levels and greater atherosclerotic burden. By comparison, women typically accumulate more subcutaneous fat, which is less directly implicated in metabolic dysfunction and vascular pathology (^29^).

Inflammatory Profiles: Sex-based differences in inflammatory mediators also contribute to divergent cardiometabolic risk. Women tend to have increased levels of adiponectin, an anti-inflammatory adipokine that enhances insulin sensitivity, whereas men have increased levels of proinflammatory cytokines, which are associated with endothelial dysfunction and atherosclerotic progression (^30^).

Together, these sex-specific physiological and biochemical factors may explain why the TyG index demonstrates enhanced predictive power for SA in women. Importantly, our findings highlight the need for sex-sensitive risk assessment tools and support the integration of the TyG index into clinical algorithms for the early detection and prevention of cardiovascular disease, particularly in female populations.

### Strengths and limitations

The strength of this study lies in its large population-based sample, which enhanced the reliability of its findings. Unlike other studies in which participants were selected from patients visiting hospitals or clinics for treatment, our participants were more representative of the general population.

However, this study has certain limitations. First, the cross-sectional study design did not allow the assessment and determination of a causal relationship between the TyG index and SA. Second, recruiting participants from health examinations at a single center may introduce selection bias and may not represent the entire population. Third, because our participants were individuals undergoing health examinations, data on confounding factors such as physical activity, dietary habits, and socioeconomic status were not collected in the standardized questionnaire, as these items were not mandatory.

In conclusion, the TyG index, a composite marker of dyslipidemia and hyperglycemia, has significant potential as a noninvasive tool for identifying individuals at risk of subclinical atherosclerosis. Our findings suggest that individuals with TyG values exceeding the determined threshold should undergo further cardiovascular evaluation, particularly women, in whom the index exhibited greater predictive value.
